# Twenty-Meter Laser Strainmeter “Popova Isl.”

**DOI:** 10.3390/s24175788

**Published:** 2024-09-05

**Authors:** Mikhail Bolsunovskii, Grigory Dolgikh, Stanislav Dolgikh, Vladimir Chupin, Viacheslav Shvets, Sergey Yakovenko

**Affiliations:** 1 Institute of Automation and Control Processes FEB RAS, 690041 Vladivostok, Russia; bolsunovsky.ma@poi.dvo.ru; 2 V.I. Il’ichev Pacific Oceanological Institute FEB RAS, 690041 Vladivostok, Russia; dolgikh@poi.dvo.ru (G.D.); chupin@poi.dvo.ru (V.C.); vshv@poi.dvo.ru (V.S.); ser_mail@poi.dvo.ru (S.Y.)

**Keywords:** laser strainmeter, Michelson interferometer

## Abstract

This paper describes the design and principle of operation of a 20 m laser strainmeter of unequal-arm type created on the basis of a Michelson interferometer and frequency-stabilized helium–neon laser. The interferometry methods used allow the measurement of the displacement of an Earth’s crust section on the base of the laser strainmeter with an accuracy of 30 pm in the frequency range from 0 (conventionally) to 1000 Hz. This laser strainmeter, when connected to an accurate time system providing an accuracy of 1 μs, should structurally become a part of the laser interferometric seismoacoustic observatory, consisting of spatially separated laser strainmeters installed in various regions of Russia.

## 1. Introduction

**Figure 1:**
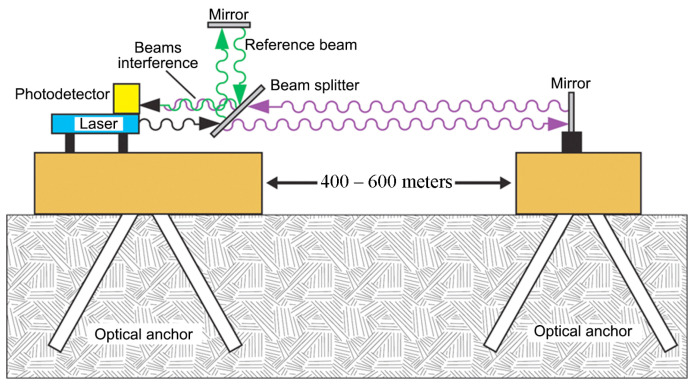
Structure diagram of a long base laser strainmeter of unequal-arm type.

To solve various problems of geophysics, many scientific organizations around the world in different years have created a large number of installations based on interferometers, which allow accurate measuring of the Earth’s crust deformations using laser interference methods. Such installations are usually called interferometer–strainmeters, or laser deformation meters (laser strainmeters). Laser strainmeters, which allow the measurement of lithospheric tides and slow deformations of the Earth’s crust in seismically hazardous areas, usually have measuring arm lengths from 50 to 800–900 m and are called long base laser strainmeters (LSM). There are both equal-arm and unequal-arm LSMs. The design of such strainmeters is similar. Figure 1 shows the most widely used structure diagram of an unequal-arm LSM.

In 1965, Vali, Krogstad, and Moss created a prototype of the first long base laser strainmeter based on a Michelson interferometer [1]. Experimental studies carried out using this laser strainmeter have provided valuable long-term measurements of variations in microdeformations of the Earth’s crust. The Vali laser strainmeter has proven its efficiency, which led to the creation at the University of California of a surface laser strainmeter based on an unequal-arm Michelson interferometer with a measuring arm length of 1000 m [2].

In the 20th century, there were several dozen laser strainmeters in the world. They can be divided into two classes: equal-arm and unequal-arm types. The optical part of laser strainmeters is built on the basis of Michelson or Fabry–Perot interferometers with practicable modifications [3,4]. Research on laser strainmeters was carried out in two directions: studying the capabilities of laser strainmeters in recording geophysical processes in a wide frequency range and studying new phenomena of low- and ultralow frequency ranges.

Currently, for recording the geodynamic, seismic, geophysical, and planetary processes of a wide range of frequencies, the most accurate means of observation are laser interferometric measuring systems. Mobile, portable, and stationary laser interferometer–strainmeters are used for measurements [5,6,7,8]. Some mobile laser strainmeters use a three-mirror interferometer design, which simplifies installation and configuration at observation sites. In stationary laser strainmeters, the optical design is based on Michelson or Fabry–Perot interferometers of equal- and unequal-arm types. Stationary laser interferometer–strainmeters are located in Russia, Japan, the USA, Italy, and other countries. The majority of interferometers are installed in the most geodynamically active regions. The measuring arms of interferometers generally do not exceed 100 m. Thus, the SAI MSU created a laser interferometer–strainmeter with a measuring arm length of 75 m; it is located in the Elbrus region [9]. In Japan, a laser strainmeter with a measuring arm length of 100 m is installed in the Kamioka mine [5,6]. There, in August 2016, a laser interferometer with a measuring arm length of 1500 m was launched in the tunnel of the KAGRA gravitational wave telescope. This device measures variations in the distance between retroreflectors installed in two vacuum chambers at a distance of 1500 m from each other [10]. In Germany, two north–south and west–east laser strainmeters were installed at the Geodynamic Observatory Moxa in 2011 in addition to the quartz strainmeters. The length of the measuring arms of these strainmeters is 100 m [11]. In California, a laser strainmeter with a length of about 500 m was installed in the Pinon Flat observatory in a tunnel at a depth of 25 m. This device is installed not far from the seashore and allows you to register not only seismic vibrations, but also microdeformations of the Earth’s crust caused by surface sea waves [7]. At the Talaya complex station (Baikal region), a laser interferometer–strainmeter with a measuring arm length of 100 m is installed in a 90 m adit [12]. Two laser strainmeters were created not far from Moscow, Russia. One, with a measuring arm length of 100 m, is installed in Fryazino, the second geophysical station in the Moscow region. With their help, atmospheric–lithospheric disturbances and the relationship between the cyclonic activity of the atmosphere and the seismicity of the Earth were studied [13]. Laser interferometer–strainmeters installed in mines and adits are intended not only for studying geophysical processes, but also for monitoring the seismic situation at installation sites [14,15].

**Figure 2:**
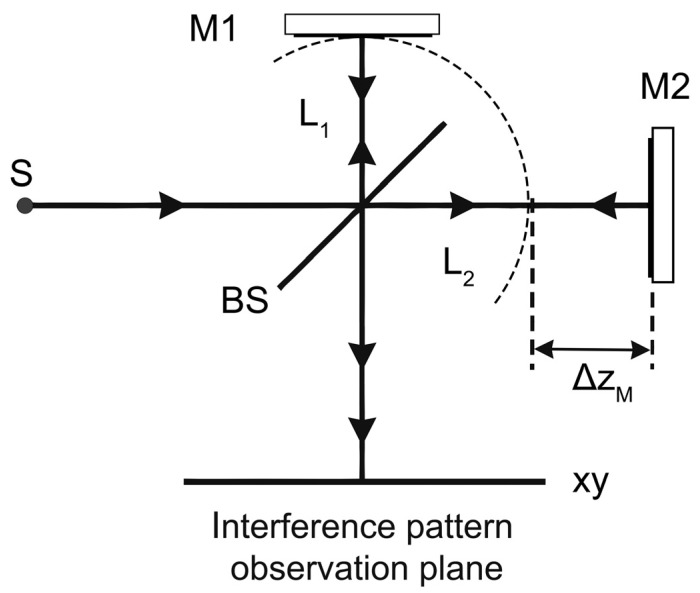
Optical scheme of Michelson interferometer: S—light source, BS—dividing plate, M1 and M2—flat mirrors, ${\Delta z}_{M}$—difference of the interferometer arm lengths.

A typical laser strainmeter consists, as we can see in Figure 1, of two bases, one of which is equipped with a laser, a dividing plate, and a mirror, limiting the reference arm of the interferometer. On the second base, there is a reflector mirror, which forms the measuring arm. This scheme is a complete functional copy of the scheme in Figure 2. The interfering rays enter into the photodetector and then into the recording equipment, which calculates the difference in the path of the rays in the interferometer. Optical anchors have a length of 20–25 m and provide a reliable connection with rock at the installation site, and also temperature decoupling from the top layer of the soil.

All laser strainmeters have virtually the same structural elements. All of them, as a rule, include polaroids, laser radiation modulation devices, one or more detectors, devices for capturing and transmitting the output signal, a laser, a reference mirror, and a dividing plate (cube). We have developed and used various laser strainmeters, with the help of which unique results were obtained in geophysics, hydroacoustics, and oceanology [16,17,18,19].

This paper describes the operating principle of a twenty-meter laser strainmeter installed on Popova Island in the Sea of Japan, which became a part of a multifunctional deformation antenna focused on the recording and direction finding of various signal sources in the infrasound and sound ranges.

## 2. Block Diagram of a Single-Axis Laser Strainmeter

**Figure 3:**
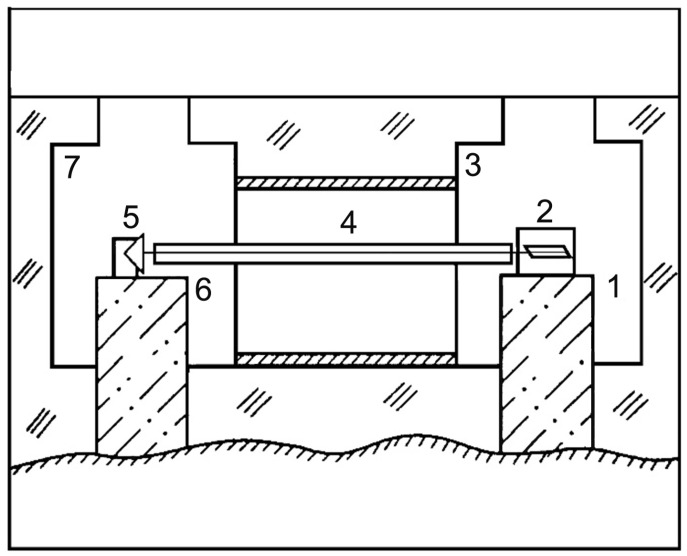
Block diagram of the single-axis-type laser strainmeter “Popova Isl.”, unequal-arm version. 1, 6—granite (concrete) blocks, 2—central interference unit, 3, 7—underground hydrothermally insulated laboratory rooms, 4—optical light guide, 5—corner reflector.

Figure 3 shows a block diagram of the “Popova Isl.” laser strainmeter of the single-axis type of the unequal-arm version, the optical elements of which are located on two blocks, rigidly connected to the elastic medium (the Earth’s crust). The optical scheme of the laser strainmeter is based on the principle of the unequal-arm Michelson interferometer with a frequency-stabilized helium–neon laser as a light source. The main interference unit of the interferometer, consisting of a laser, a dividing plate (cube), mirrors on piezoceramic cylinders, a collimator, and adjustment mechanisms, is located on a powerful granite–concrete block of 1.5 × 1.5 × 3.0 m, which is rigidly connected to the main rocks of the upper layer of the Earth’s crust. The main interference unit of the laser strainmeter (2) and the corner reflector (5) are located in separate hydrothermally insulated rooms, in which the maximum temperature variations reach values of about 0.1 K. The optical light guide (4), which is used in air-filled or vacuum versions, is assembled from stainless steel pipes with an internal diameter of about 10 cm.

The readings of the laser strainmeter are affected by errors caused by variations in temperature, pressure, and humidity. Variations in temperature, pressure, and humidity affect the stability of the operating laser frequency and the linear dimensions of the interferometer structural elements. Long-term frequency stability in the tenth to eleventh decimal places (short-term frequency stability is an order or two higher) fully ensures the specified measurement accuracy (30 pm).

Below, we will consider in more detail the measurement errors of the unequal-arm laser strainmeter caused by variations in temperature, humidity, and pressure. When using laser strainmeters of the surface version, the reflectors and the interference unit with the laser are located in thermally insulated rooms, in which multi-day temperature variations amount to about 0.01 K. In the main interference unit, consisting of plane-parallel mirrors, piezoceramic cylinders, and an invar plate, the error is $\Delta L=\pm 0.2\times 10^{-9}$ m. The total error caused by changes in temperature, pressure, and humidity in the air spaces of the interferometer is $\Delta L=\pm 1.1\times 10^{-10}$ m. When working on any strainmeter with an air-filled or vacuum pipeline, it is necessary to maintain the pressure in the light guide with an accuracy of $\Delta P=2\times 10^{-10}/0.4\times 10^{-6}=5\times 10^{-4}$ mm Hg to ensure a sensitivity of $\Delta L/L\approx 2\times 10^{-10}$. Pressure variations in the pipe (10^−4^ mm Hg) ensure more than necessary measurement accuracy. Let us estimate the temperature that will not affect the accuracy of measurements at $P=10^{-4}$ mm Hg and $\varepsilon =\delta L/L=2\times 10^{-10}$; $\delta L/L=\alpha n_{T}=2\times 10^{-10}\Delta T$; $\Delta T=\delta L/\left(L\times 2\times 10^{-6}\right)=1$ deg. Maintaining a temperature equal to const±0.1 K allows us to exclude the effect of this error on the accuracy of measurements. The change in the pipe length due to a change in atmospheric pressure will be ΔL=ΔFL/AY (where ΔF is the change in the force of atmospheric pressure at the end of the pipe due to a change in atmospheric pressure; *L* is the length of the pipe (20 m); A is the cross-section area of the pipe walls; $\Delta F=\pi r^{2}\Delta P$; *r* is the radius of the pipe; ΔP is the change in atmospheric pressure). Since $\Delta F=3.14\times 10^{6}$ dyn, L=20 m, A=19 cm^2^, $Y=2\times 10^{11}$ N/m, then $\Delta L=\pi r^{2}\Delta Pl/AY\;$ and, consequently, $\Delta L=2.2\times 10^{-10}$ m.

We should note that temperature variations in temperature-controlled chambers during the operation of laser strainmeters with shoulder lengths of 52.5, 17.5, and 20 m of a stationary, underground version in rooms with good hydro- and thermal insulation are less than 0.01 K and are caused mainly by multi-day temperature fluctuations. When studying seismoacoustic oscillations on these laser strainmeters, the measurement error is 1–2 orders of magnitude smaller and the measurement accuracy, accordingly, increases. During studies in the range of 1–10^3^ Hz, the measurement error decreases further, since variations in temperature, pressure, and humidity are of a lower-frequency nature.

The change in the distance between these blocks is measured using interference methods. Let us call such a laser strainmeter “a single-axis laser strainmeter of the classical type”. Various processes cause changes in the distance between blocks: oscillatory and wave, meteorological, geophysical, geodynamic, etc. When a laser strainmeter is located on the surface of the Earth, it can record surface Rayleigh waves and longitudinal and transverse waves. To study the features of the amplitude–frequency characteristics of a classical-type laser strainmeter, we will focus on the simplest case: recording a harmonic wave of longitudinal type propagating in the Earth’s crust. In this case, the laser strainmeter blocks are located on homogeneous medium. Let a harmonic wave of longitudinal type propagate along the axis of the laser strainmeter, which can be described by the following equation:

(1) $$u_{1}=A_{0}\cos(\omega t-kx_{1}),$$

where $u_{1}$ is the displacement of a particle of the Earth’s crust at point $x_{1}$, $A_{0}$ is the amplitude of the harmonic wave, k=2π/λ is the wave number, λ is the wavelength, ω=2πν is the cyclic frequency, ν is the wave frequency, t is the current time.

A classical-type laser strainmeter, when an elastic wave propagates through the medium at its location, will record a displacement equal to the change in the distance between the blocks:

(2) $$\Delta L=x_{2}-x_{1}=2A_{0}\sin\left(\frac{kL}{2}\right)\sin\left(\omega t-kx_{1}-\frac{kL}{2}\right)$$

where L is the length of the measuring arm of the strainmeter (the distance between the blocks of the laser strainmeter ), $x_{1}$ is the coordinate of the first block of the strainmeter, $x_{2}$ is the coordinate of the second block of the strainmeter, $x_{2}=x_{1}+L$, $u_{2}=A_{0}\cos(\omega t-kx_{2})$. As we can see in Equation (2), the wave amplitude $A_{reg}$ recorded by the laser strainmeter depends on $A_{0}$, L, and λ.

**Figure 4:**
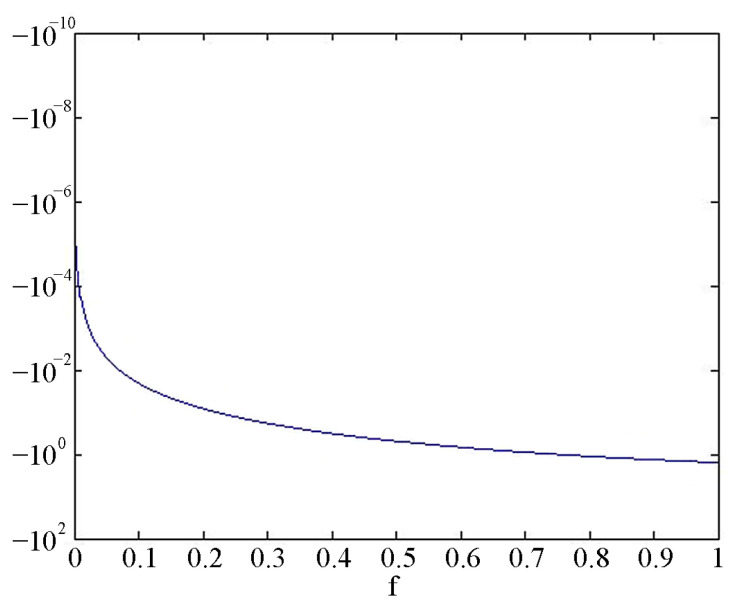
Amplitude–frequency response of a classical-type single-axis laser strainmeter with measuring arm length of 20 m in the infrasound region (0–1 Hz).

**Figure 5:**
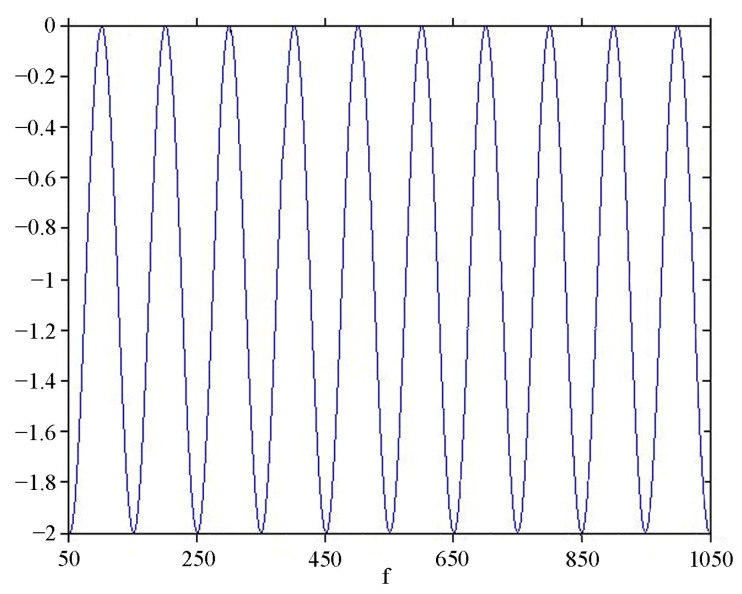
Amplitude–frequency response of a classical-type single-axis laser strainmeter with measuring arm length of 20 m in the infrasound region (50–300 Hz).

Next, we will consider the change in the amplitude–frequency characteristic of a classical-type single-axis laser strainmeter in the frequency range from 0 to 300 Hz when recording a longitudinal harmonic wave. Let the length of the measuring arm of the laser strainmeter be 20 m, the amplitude of the wave be 1, and its speed be 2000 m/s. Figure 4 shows the amplitude–frequency characteristic of a single-axis laser strainmeter of the classical type with a measuring arm length of 20 m in the infrasound region (0–1 Hz), where the frequency in Hz is plotted along the abscissa axis, and $A_{reg}$ is plotted along the ordinate axis. As the measuring arm of the laser strainmeter increases, its sensitivity in the infrasound region increases. In the high-frequency region, starting from the frequency of about 50 Hz, the amplitude–frequency characteristic of this laser strainmeter changes according to the harmonic law (beat zone); see Figure 5. That is, the device at some frequencies outputs a double amplitude of the wave (modulo $\left|A_{reg}\right|=2A_{0}$), and at some it outputs 0 instead of the real amplitude equal to 1, i.e., in the beat zone, it is difficult to carry out registration due to periodic changes in the amplitude–frequency characteristics. It is impossible to theoretically calculate and determine experimentally all the features of specific laser strainmeters due to the heterogeneous structure of the Earth’s crust in their location areas.

## 3. The Principle of Displacement Recording on the Base of a Laser Strainmeter

To record displacements, this laser strainmeter uses the so-called modulation method. The essence of this method is that the path difference between the interfering beams is changed within small limits according to the periodic law and, thereby, the light intensity at the output of the interferometer is modulated. Let us write the path difference in the form $\Delta =\bar{\Delta }+\Delta_{0}\sin\omega t$. Then, for the phase difference, we have, respectively, $\delta =\bar{\delta }+\delta_{0}\sin\omega t$. The equation for intensity distribution in this case has the following form:

(3) $$J=J_{0}\cos^{2}\frac{\delta }{2}=J_{0}(1+\cos\delta )=J_{0}+J_{0}\cos(\bar{\delta }+\delta_{0}\sin\omega t)\;=J_{0}+J_{0}\cos\bar{\delta }\cos(\delta_{0}\sin\omega t)-J_{0}\sin\bar{\delta }\sin(\delta_{0}\sin\omega t).$$

**Figure 6:**
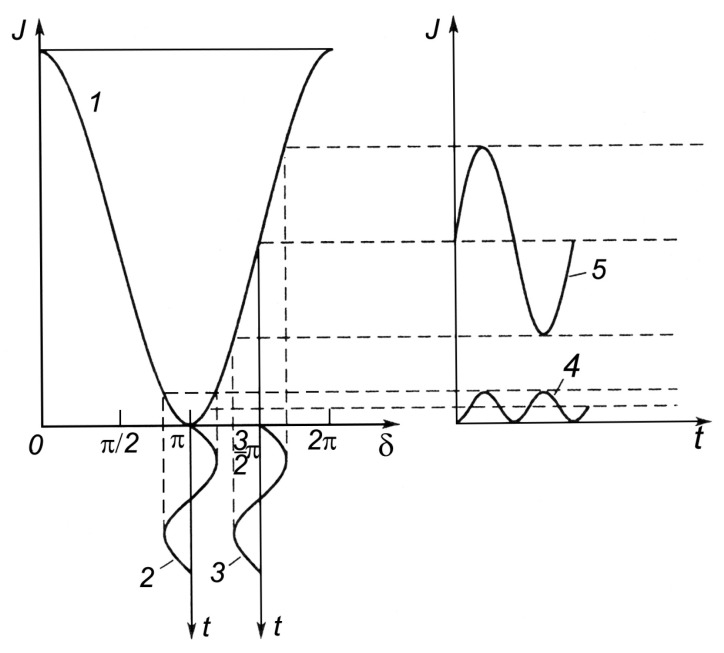
Graphical representation of the modulation method.

Figure 6 demonstrates the modulation method. The change in the resulting intensity *J* as a function of the phase difference *δ* is represented by curve 1. The dependence of the change in the phase difference on time *t* near $\bar{\delta }=\pi$ and $\bar{\delta }=2\pi$ is represented by curves 2 and 3. Then, with periodic phase changes near the indicated values, the phase at the interferometer output is represented by curves 4 and 5.

When $\bar{\delta }=\pi$, the variable component of the intensity, and, accordingly, of the signal, practically does not contain the first harmonic (curve 4). When $\bar{\delta }=3\pi /2\;$, the variable component of the signal can be expressed with great accuracy by the first harmonic of the Fourier expansion (curve 5). If we use a narrow-band electric filter to isolate the first harmonic, then by the absence of the signal, we can determine with high accuracy the moment of pointing to the minimum in the interference pattern. The first harmonic is absent not only in the minimum intensity (curve 1), but also in the maxima.

**Figure 7:**
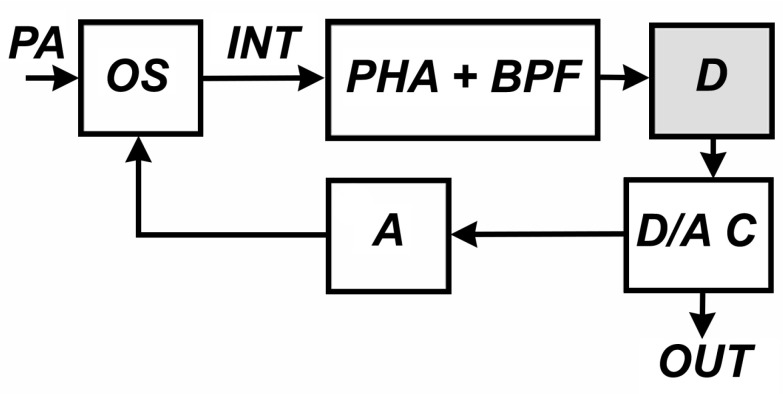
Functional diagram of the laser strainmeter registration system. PA—perturbation influence, OS—optical system, PHA + BPF—photocurrent amplifier and bandpass (or resonant) filter, D—detector, D/A C—digital-to-analog converter, A—amplifier, OUT—output signal.

During the 1990–2000s, using compensation measurement methods, we developed recording systems for laser strainmeters of various modifications. In recent years, modifications of recording systems based on modern microcontrollers have been created. Figure 7 shows a basic functional diagram for such systems.

**Figure 8:**
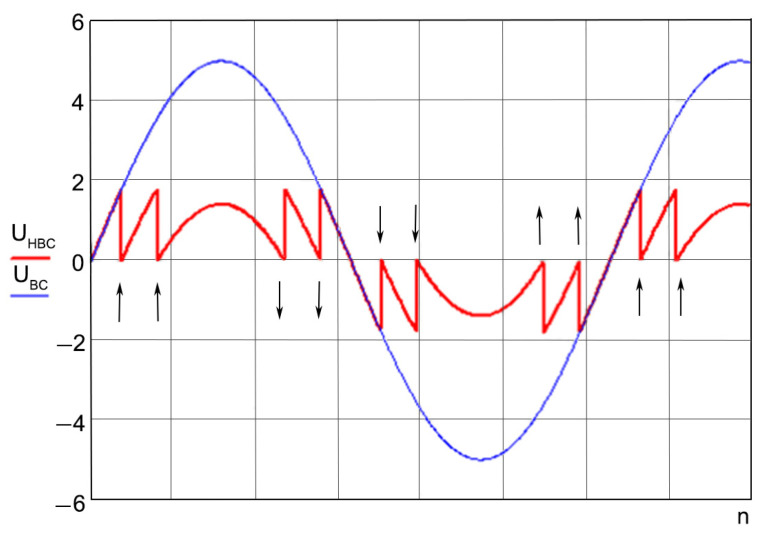
The procedure of restoring the output signal from its pieces. U_HBC_—unrestored voltage, U_BC_—restored voltage. The arrows indicate the direction of the offset of the U_HBC_ graph.

Here, the external influence, PA, changes the state of the interference pattern that is formed in the optical system, OS, of the interferometer. The introduction of harmonic modulating oscillations into the system is carried out by a piezoceramic cylinder with a mirror mounted on it. The mirror, which, according to the optical design of the laser strainmeter, forms a support arm, is mounted on the second piezoceramic cylinder. The compensating influence necessary to return the interference pattern to one of the extreme values is provided by the D/A C—A chain. The PHA-BPF block plays the role of a narrow-band filter, as indicated in the description of the method, and the detector, D, allows curves 4 and 5 in Figure 6 to be presented in a form convenient for processing. The output signal, proportional to the path difference of the beams in the laser strainmeter, is the output voltage of the D/A C. In the general case, the magnitude of the compensating voltage supplied to the amplifier, A, and affecting the optical system through the mirror of the strainmeter support arm may be greater than the maximum permissible for a piezoceramic compensator cylinder. Therefore, from time to time, when a certain threshold value is exceeded, a transition is made to the neighboring extremum of the interference pattern, and the output voltage of the D/A C is reset to zero. The output signal is, thus, represented by pieces of the output signal curve (Figure 8). The operation of restoring the output signal is a common procedure for laser strainmeters using modulation compensation methods. For restoration, we use the following expression:

(4) $$u(n,t)=u_{cб}\sum_{n=1}^{k}S_{n}+u_{\begin{array}{c} car \end{array}}(t)$$

where *n* is the number of recorded transitions of the “scale” from the maximum or minimum value to zero, *S_n_* is the sign of the transition, and *u_car_*(*t*) is the instantaneous value of the output voltage in the considered piece of recording.

The most important unit of the registration system for implementing the modulation method is the controller–detector. The main task of the controller–detector is to calculate the value of the output voltage of the recording system, which, after applying it to the actuator (device), will ensure the return of the interference pattern of the device to its extreme position. The property of the output signal of extreme control systems with synchronous detection is used to change the phase of the output signal of the controlled object by 180°; the controller acts as a relay regulator.

**Figure 9:**
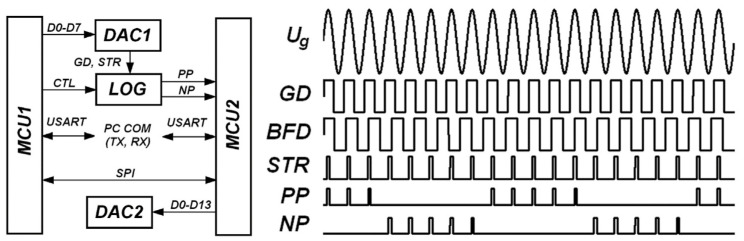
Block diagram of the controller and timing diagram demonstrating the operation of its constituent units. MCU1, MCU2—microcontrollers; LOG—logical block; DAC1—resistive two-stage 8-bit DAC; DAC2—executive DAC, 12–14 bits; Ug—search sinusoidal signal; GD and BFD—amplified and limited search and output signal of the object; STR—measuring strobe; PP and NP—signal pulses.

The controller includes two microcontrollers (Figure 9). The MCU1 microcontroller communicates with a personal computer via a USART (universal serial transceiver) connected through a level converter (for example, MAX232). The data transmitted to the MCU1 microcontroller sets the operating modes of the controller in general. The MCU2 microcontroller, through its USART, transmits the output signal of the controller–detector to the recording computer, but uses the RS-485/422 interface for this. Data exchange between MCU1 and MCU2 goes on via the SPI bus.

The controller search device is a sinusoidal voltage generator. To improve the quality of operation under conditions of the control object characteristic drift, a signal of a complex form can be generated, which is the sum of the main signal and its second harmonic. The generator is based on an 8-bit two-stage resistive digital-to-analog converter (DAC1), which is controlled through the microcontroller port MCU1 (ATmega16). A period of time equal to the period of the output voltage created by the generator is divided into N equal parts. For each kth sample (*k* = 0.... N), the value of function is calculated:

(5) $$S\left(k\right)=\frac{\sin(2\pi k/N)+1}{2},$$

**Figure 10:**
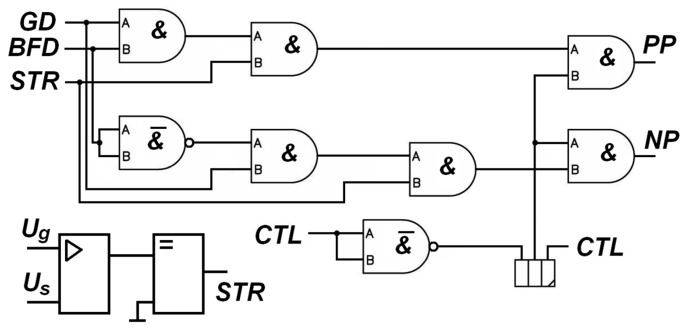
Controller–detector logic unit. GD and BFD—amplified and limited search and output signal of the object; STR—measuring strobe; PP and NP—signal pulses; CTL—signal diagnostic subsystem of the controller; U_g_— search signal of the system; U_s_—shifted.

The binary value of the function *S*(*k*), rounded to the nearest integer value, is supplied to the inputs of the DAC every time an interrupt occurs in the T0 timer of the MCU1 microcontroller, thus forming a voltage of $U_{g}\left(t\right)=U\sin\omega t$, which is then filtered from the constant component and shifted by U_s_. The resulting voltage is supplied to the comparator, and the output voltage of the comparator STR (signal pulse) is sent to the logic unit of the controller (detector). The GD and BFD signals are obtained by clipping amplification from the voltages U_g_ (search signal of the system) and U_BF_ (reaction signal of the regulated object). The LOG detector pulses (PP and NP) tell the MCU2 microcontroller to increment or decrement the value output to DAC2 (which generates the recording system output). The CTL signal is generated by the diagnostic subsystem of the controller and blocks the operation of the detector in the event of a malfunction. The logic design stipulates the possibility of using either a high- or low-level CTL signal when an alarm occurs in the recording system. The above is shown in Figure 10.

The detector signals are connected to each other through the following correlations:

(6) $$\begin{array}{l} UG=U_{g}(t)=\sin(\omega t),\\ UGI=U_{gi}(t)=-U_{g}(t)=-\sin(\omega t),\;GD=U_{GD}(t)=\frac{1}{2}sign(U_{g}(t))+\frac{1}{2},\\ BF=U_{BF}(t)=\sin(\omega_{1}t+\varphi ),\;\varphi =const,\;s=const\\ BFS=U_{BFS}(t)=\left[\frac{1}{2}sign((U_{BF}(t)-s)+\frac{1}{2}\right]\left[U_{BF}(t)-s\right],\\ BFSD=U_{BFSD}(t)=sign(U_{BFS}(t)),\;BFD=U_{BFD}(t)=\frac{1}{2}sign(U_{BF}(t))+\frac{1}{2},\\ TS=U_{TS}(t)=\left[\frac{1}{2}sign((U_{g}(t)-s)+\frac{1}{2}\right]\left[U_{g}(t)-s\right],\\ STR=U_{STR}(t)=sign(U_{TS}(t)),\\ ITS=U_{ITS}(t)=\left[\frac{1}{2}sign((U_{gi}(t)-s)+\frac{1}{2}\right]\left[U_{gi}(t)-s\right],\\ ISTR=U_{ISTR}(t)=sign(U_{ITS}(t)),\\ PP=U_{PP}(t)=\left[U_{STR}(t)⊗U_{BFSD}(t)\right]⊗U_{CTL}(t),\\ NP=U_{NP}(t)=\left[U_{ISTR}(t)⊗U_{BFSD}(t)\right]⊗U_{CTL}(t). \end{array}$$

**Figure 11:**
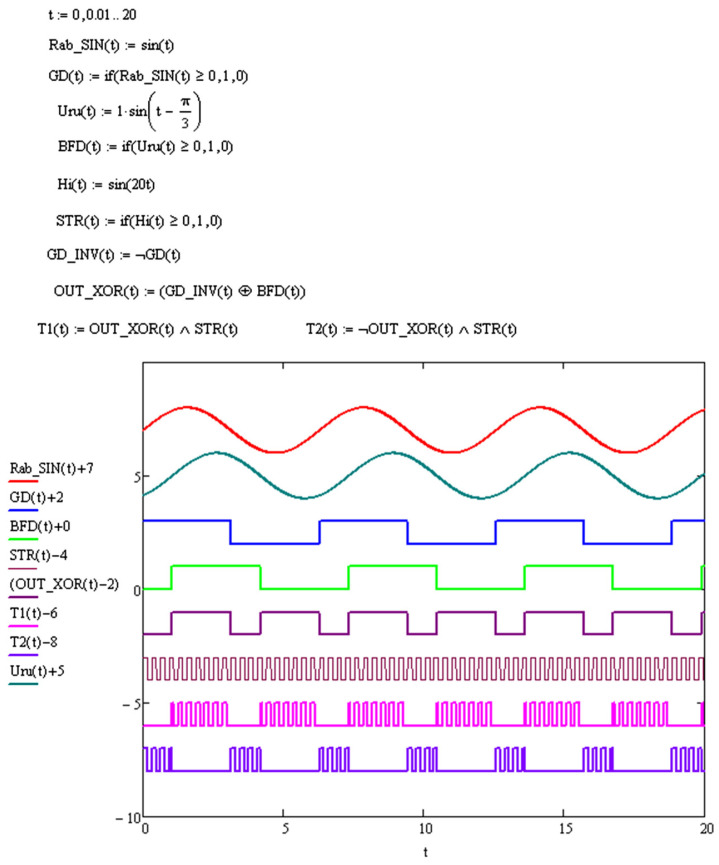
Implementation of the given controller–detector signals in the Mathcad system.

**Figure 12:**
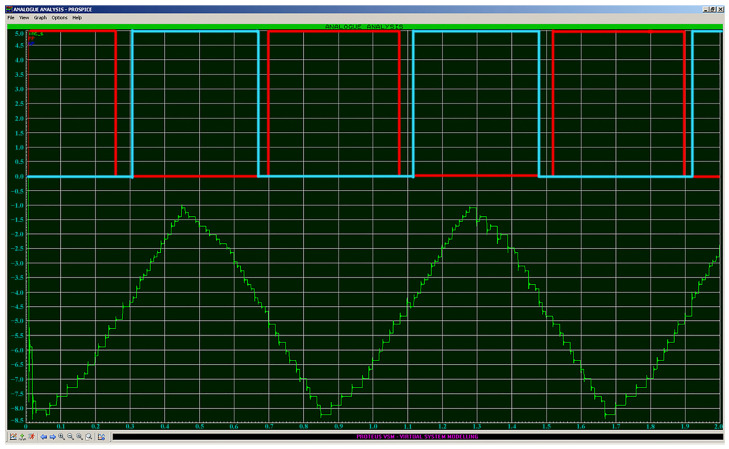
Visualization of the output signal of the phase detector when two signals with similar frequencies are supplied. Red—PP signal; Blue—NP signal; Green—output signal.

For example, for the case when the phase difference of the signals received by the controller is −π/3, in the simulation in the Mathcad system, we get the following (Figure 11):

The phase shift by an angle of −π/3 leads to the fact that the difference in the number of counted pulses for signals T1 and T2 (pink and purple graphs in Figure 11) becomes positive, which explicitly determines the position of the operating point relative to the extremum and the necessary direction for compensating impact. With a phase difference of +π/3, the situation will be the opposite: the difference in the counted pulses T1 and T2 will be negative. Figure 12 shows a graph of the actual operation of the detector. The input signals are two sinusoidal signals with frequencies of 100 and 100.01 kHz. The red and blue signals correspond to the PP and NP signals, which, as stated above, are defined as

(7) $$PP=U_{PP}(t)=\left[U_{STR}(t)⊗U_{BFSD}(t)\right]⊗U_{CTL}(t),\;NP=U_{NP}(t)=\left[U_{ISTR}(t)⊗U_{BFSD}(t)\right]⊗U_{CTL}(t)$$

**Figure 13:**
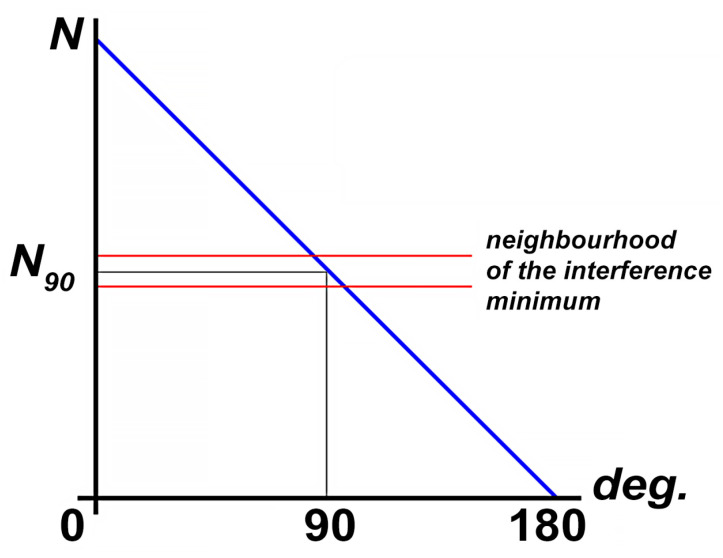
Static characteristics of the phase detector. N—difference of counted pulses T1 and T2, N_90_—number of pulses counted at phase shift equal to 90°.

**Figure 14:**
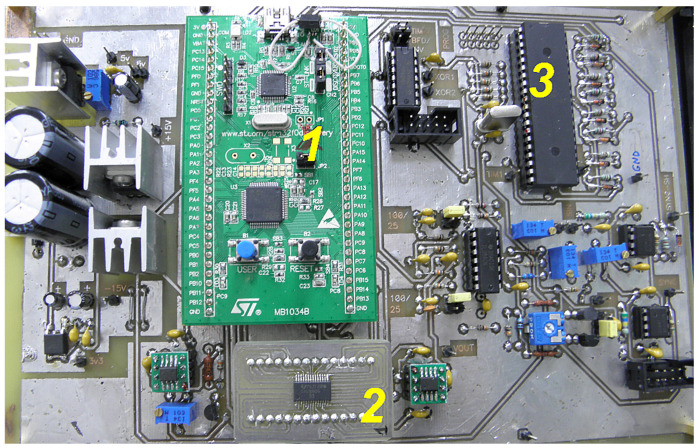
One of the implementations of the controller–detector (Laboratory of Physics of Geospheres). 1—MCU1 based on the STM32F0 Discovery board, 2—bipolar digital-to-analog converter based on DAC8806, 3—MCU2 ATmega16.

Thus, we obtain the static characteristics of the phase detector necessary for implementing detection using the modulation method (Figure 13):

The controller described above is used in laser strainmeters that are part of the seismoacoustic–hydrophysical complex of POI FEB RAS. The main technical characteristics are as follows: search signal frequency—25 or 100 kHz; the operating frequency range is up to 1 kHz; the maximum data transfer rate is 1820 kB/s; and the accuracy of measuring the path difference of the interferometer beams is 0.03 nm (with a DAC2 capacity of 14 bits and measuring arm length of 20 m). Figure 14 shows one implementation of the controller–detector.

## 4. Test Trials

**Figure 15:**
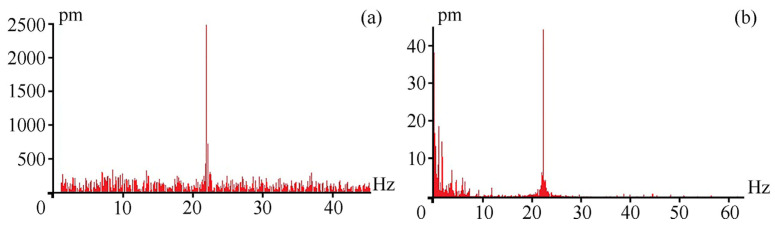
Spectra obtained from processing the laser strainmeter record at the moment of operation of the hydroacoustic emitter at the frequency of 22 Hz.

The laser strainmeters located at Shultz Cape in the Sea of Japan [8] were designed on the same principle, using similar optical elements, frequency-stabilized lasers, and the principle of recording displacements at the base of the laser strainmeter, on which unique results in various frequency ranges (from recording tones and overtones of the Earth’s eigen oscillations to recording high-frequency signals of marine origin) were obtained. All the instruments have undergone testing related to recording known signals, which primarily include signals generated in water by low-frequency hydroacoustic emitters and signals generated in the Earth’s crust by earthquakes. In our case, Figure 15a shows the spectrum obtained from processing the laser strainmeter record at the moment of operation of a low-frequency hydroacoustic emitter, which created hydroacoustic oscillations in the water at the frequency of 22 Hz [20], located at the distance of about 5 km from the strainmeter. Figure 15b shows the spectrum obtained from processing the laser strainmeter record at the moment of operation of a low-frequency hydroacoustic emitter, which created hydroacoustic oscillations in the water at the frequency of 22 Hz, located at the distance of about 34 km from the strainmeter.

**Figure 16:**
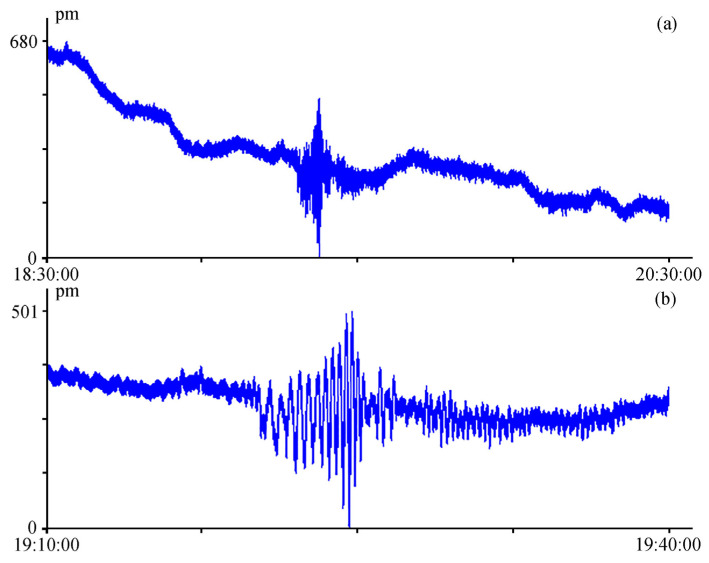
Registration of the Kamchatka earthquake on 17 August 2024 by the twenty-meter laser strainmeter (UTC time).

As we can see from the provided spectra, the laser strainmeter successfully records seismoacoustic signals with an amplitude of about 44 pm. From the provided spectrum, shown in Figure 15b, we can conclude that the instrument can record signals with smaller amplitudes. In a lower frequency range, Figure 16 shows, for example, a record of the laser strainmeter that recorded an earthquake with a magnitude of 7 that occurred in the Kamchatka area. The earthquake occurred on 17 August 2024, in the point with coordinates 52.924° N and 160.141° E, at the depth of 29 km. The epicenter of the earthquake was 100 km away from the Kamchatka Peninsula at the distance of about 2370 km from the instrument installation site. Figure 16a shows a two-hour record of the laser strainmeter at the time of earthquake registration, and Figure 16b shows an enlarged fragment of this record.

## 5. Conclusions

We have described the principle of operation of the twenty-meter laser strainmeter located on Popova Island, in the Sea of Japan, in the place with coordinates 42.98° N, 131.72° E, which, structurally, is a part of the laser interferometric seismoacoustic observatory. The observatory also consists of 52.5 m and 17.5 m laser strainmeters located at Shultz Cape in the Sea of Japan [8]. The main purpose of this observatory is registration, direction finding, and identification of infrasound signals generated by various geodynamic processes in the “atmosphere-hydrosphere-lithosphere” system. The conducted test trials allow us to hope that the laser strainmeter will be able to register signals with amplitudes less than 10 pm, which will permit us to track informative deformation disturbances at virtually any planetary distance.

The main technical characteristics of this laser strainmeter are as follows: length of the measuring arm—20 m, operating frequency range—from 0 to 1000 Hz, accuracy of measuring displacements on the device base—30 pm.

## Data Availability

Third-party data were used. Restrictions apply to the availability of these data.
